# Emerging Therapeutic Strategies to Restore Regulatory T Cell Control of Islet Autoimmunity in Type 1 Diabetes

**DOI:** 10.3389/fimmu.2021.635767

**Published:** 2021-03-18

**Authors:** Victoria Volfson-Sedletsky, Albert Jones, Jaileene Hernandez-Escalante, Hans Dooms

**Affiliations:** ^1^ Arthritis and Autoimmune Diseases Research Center, Rheumatology Section, Department of Medicine, Boston University School of Medicine, Boston, MA, United States; ^2^ Department of Microbiology, Boston University School of Medicine, Boston, MA, United States

**Keywords:** type 1 diabetes (T1D), immunotherapy, autoimmune disease (AD), T cells, Tregs (regulatory T cells), nanotechnology/nanomaterials, antigen-specific therapies, cell-based therapeutics

## Abstract

Despite many decades of investigation uncovering the autoimmune mechanisms underlying Type 1 Diabetes (T1D), translating these findings into effective therapeutics has proven extremely challenging. T1D is caused by autoreactive T cells that become inappropriately activated and kill the β cells in the pancreas, resulting in insulin insufficiency and hyperglycemia. A large body of evidence supports the idea that the unchecked activation and expansion of autoreactive T cells in T1D is due to defects in immunosuppressive regulatory T cells (Tregs) that are critical for maintaining peripheral tolerance to islet autoantigens. Hence, repairing these Treg deficiencies is a much sought-after strategy to treat the disease. To accomplish this goal in the most precise, effective and safest way possible, restored Treg functions will need to be targeted towards suppressing the autoantigen-specific immune responses only and/or be localized in the pancreas. Here we review the most recent developments in designing Treg therapies that go beyond broad activation or expansion of non-specific polyclonal Treg populations. We focus on two cutting-edge strategies namely *ex vivo* generation of optimized Tregs for re-introduction in T1D patients vs direct *in situ* stimulation and restoration of endogenous Treg function.

## Introduction

T1D is an autoimmune disease resulting in loss of the insulin-producing β cells in the pancreas, leading to hyperglycemia. Although T1D can appear at any age, it is mostly prevalent in children and is considered to be one of the most common childhood chronic diseases, with an increasing incidence of 3-4% over the past three decades (1). A loss of immune regulation caused by a combination of underlying genetic susceptibilities and as yet undefined environmental factors enables autoreactive CD4+ and CD8+ T cells to destroy the pancreatic β cells (2–5). Hence, the ultimate goal for the treatment of T1D is to restore the defects in immune regulation to achieve durable tolerance to islet autoantigens. Regulatory T cells (Tregs) are an extremely attractive cell population to utilize for restoring tolerance in T1D since these cells are known to be functionally deficient in T1D, and this defect contributes to disease progression (6). In the NOD mouse model of T1D, as well as in T1D patients, deficiencies in the Treg TCR repertoire (7), the IL-2/IL-2Rα pathway (8–11) and suppressor mechanisms such as CTLA-4 (12) may all contribute to reduced Treg functionality. Animal studies have demonstrated that bolstering the Treg compartment through adoptively transferring polyclonal or islet antigen-specific Tregs (13–16) or by administering low-dose IL-2 can prevent and reverse T1D (11, 17–19). Hence, transforming Tregs into highly efficient, targeted and localized suppressors of autoreactive T cells carries enormous potential as the *nec plus ultra* for using Tregs to cure T1D and other autoimmune diseases. Here, we focus on emerging strategies with high potential for clinical translation to not just increase Tregs indiscriminately but to do so in a precisely targeted way with minimal side effects. We weigh the advantages and disadvantages of manipulating Tregs *ex vivo* to optimize their specificity and function before re-introducing the cells into patients vs approaches to directly target antigen-specific Tregs for expansion and functional enhancement in disease-relevant tissues, using cutting-edge delivery systems such as nanomaterials.

## Cell-Based Therapies Using Polyclonal Tregs

**Figure 1:**
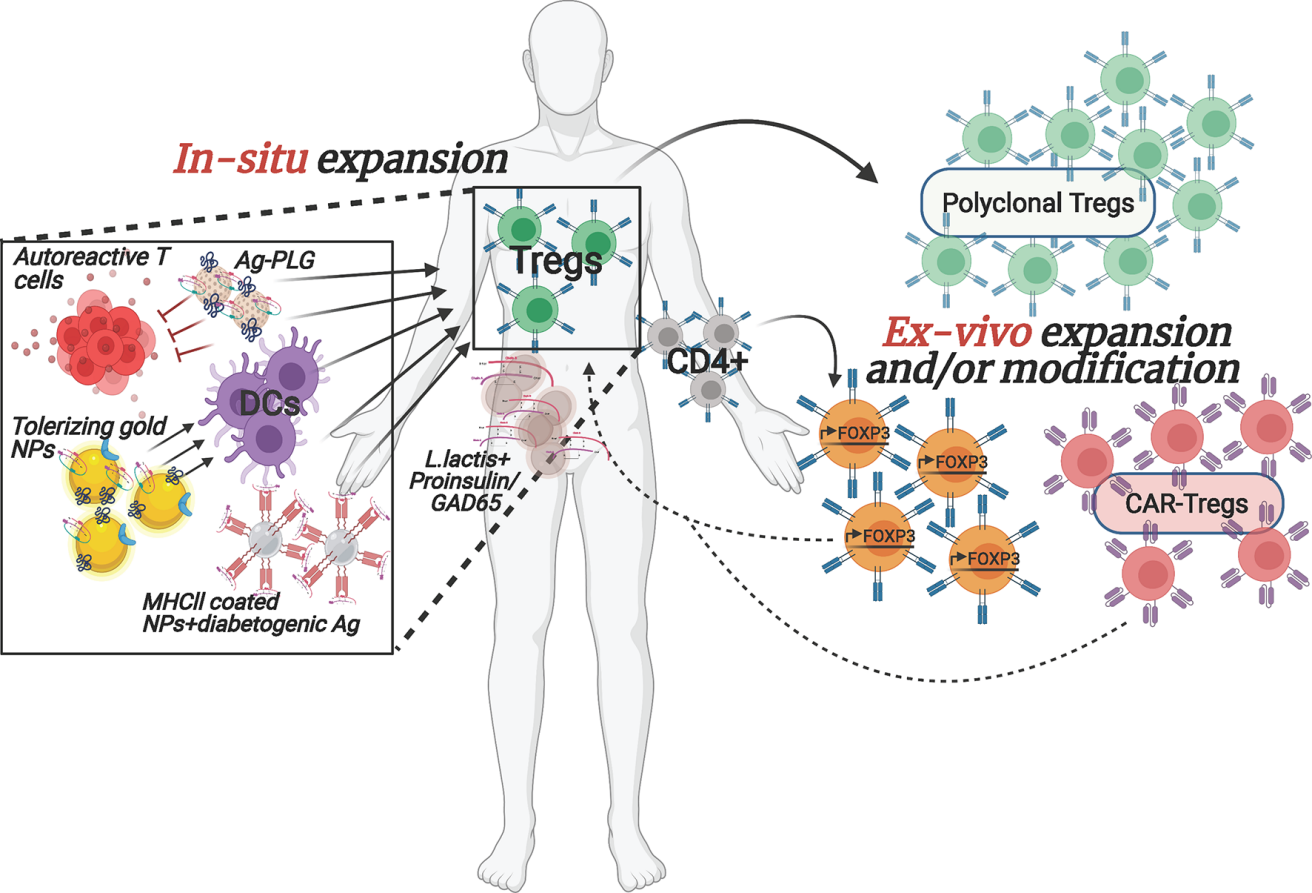
Overview of selected emerging *ex vivo* and *in situ* Treg enhancement approaches for T1D. The depicted therapeutic strategies to enhance Tregs in T1D patients are currently being implemented both pre-clinically as well as in clinical trials. The interventions are designed to (1) increase autologous Treg cell numbers, *ex vivo*, with or without modification prior to transplanting them back into the patient or (2) reinforce Tregs within the patient by stimulating/re-activating them *via* nano-particles carrying specific auto-antigens and/or by converting the pathogenic cells to protective, regulatory cell populations. Arrows denote stimulatory/activating effect.

Cell-based therapies are a new frontier in the treatment of autoimmune diseases and cancer. Although different immunosuppressants and biologic treatments have greatly improved in the past decade and are getting more efficient in ameliorating disease manifestations, cell-based therapies carry tremendous promise due to their diversified and adaptable array of therapeutic activities, and their capacity to locate to the site of lesion. In autoimmune diseases, current treatments are often alleviating symptoms and promote episodes of remission but do not fundamentally cure the disease. Cell-based approaches on the other hand hold the power to provide a cure for autoimmunity by durably establishing therapeutic cell populations in patients to tolerize and eliminate autoreactive immune cells and permanently heal tissues. Tregs, due to their documented dysfunctionality in T1D, are ideal candidates for cell-based therapies aiming to strengthen their numbers and/or function **(** **Figure 1** **)**. Increased enthusiasm for this approach stems in part from success in clinical trials in other conditions such as graft versus host diseases (GvHD), where the beneficial impact of Tregs has been shown in prophylaxis and during its chronic state (20–22). This success is even more impressive given the obstacles that accompany the use of these cells, namely their potential impurity upon isolation (e.g. contamination with effector T cells (Teff)), limited *in vitro* expansion capacity, potential to differentiate into other cells types, and their post-transplantation survival capacity. These issues will need to be resolved before becoming part of standard care.

To test Treg therapy in T1D, recent phase l clinical trials have evaluated the safety and efficacy of adoptive transfer of polyclonal Tregs (23–25), (NCT01210664, ISRCTN06128462) to reverse recent onset T1D in patients. Patients received infusions of autologous *ex vivo* expanded polyclonal Tregs (identified by the markers CD4+CD127lo/-CD25+) in several doses. Both trials showed that the treatment was well-tolerated and safe. One of the studies (25) showed that cells remained mostly true to their identity with minor surface marker changes, and with no evidence of converting into Teff cells. Approximately 3 months following the infusion, a quarter of the maximal cell number that was detected shortly after infusion could still be traced in the circulation, followed by a period of stabilization where cells could still be detected 1 year post-infusion. In terms of disease outcome, the number of participants and heterogeneity of diabetes make it hard to draw conclusions, but the trial reported several individuals with unchanged C-peptide levels while others showed decline. Of note, these results are within the expected decline compared to the natural history of the disease. The other clinical trial (23, 24) showed significantly higher C-peptide levels in treated children compared to non-treated. Moreover, some children were less dependent on exogenous insulin (24). At a 2 year follow up, some treated subjects required significantly lower doses of insulin and had higher C-peptide levels, especially children that received two doses of Tregs.

The safety and success of transferring autologous *ex vivo* expanded Tregs to recent onset T1D patients was also tested in a recent phase ll clinical trial (NCT02691247). However, even though the treatment was well-tolerated, patients failed to show improvement in C-peptide levels. It is hard to speculate at this point, whether the mixed results from these studies are due to differences in patients (e.g. age of the patient and type of islet autoantibodies present) and study design (e.g. different dosages of Tregs) or whether this treatment in its current setting is not efficient enough. Noteworthy, a new trial is currently evaluating whether results can be improved by administrating IL-2 to recipients of autologous polyclonal Tregs to improve survival and function of the transferred cells (NCT02772679). Unfortunately, early results presented at the meeting “The Promise of Interleukin-2 Therapy” in Paris 2019 indicated that patients treated with IL-2 may show increased decline of C-peptide, cautioning that significant challenges remain to use IL-2 in the clinic for T1D.

## Emerging Approaches to Generate Antigen-Specific Tregs *Ex Vivo*

Although the studies above managed to overcome some major technical hurdles, the difficulties for broad usage of adoptive transfer of polyclonal Tregs in the clinic due to their very low frequency in circulation and difficulty to expand the cells *in vitro* while maintaining their identity and functionality, still stand. In addition, some individuals may have inherent defects in their Treg population, rendering them ineffective for treatment. Importantly, preclinical studies in animal models of T1D suggest that the use of antigen-specific, rather than polyclonal, Tregs will be more efficacious in controlling the disease (13, 26–29). However, isolating sufficient numbers of islet autoantigen-specific Tregs from patients for *in vitro* expansion is even more challenging than polyclonal Tregs, since most of them are located in the target tissue, in this case the pancreas, limiting their accessibility. Moreover, extremely limited information about their TCR epitope specificity is available. Therefore, potential solutions to these obstacles are being developed. To increase the numbers of Treg cells for adoptive therapy, Honaker et al. (30). developed a method to convert autologous CD4+ effector T cells into Treg-like cells. To do so, they implemented a gene editing approach, homology-directed repair (HDR), to insert a strong promoter in the forkhead box P3 (FOXP3) locus, the master transcriptional regulator for Tregs, of polyclonal CD4+ T cells, in a strategic location that would bypass potential epigenetic silencing. This will lead to expression of endogenous FOXP3 in bulk CD4+ T cells **(** **Figure 1** **)**. These edited Tregs (edTregs) expressed many of the canonical Treg markers and were more sensitive to low doses of IL-2 compared to mock-edited cells, and edTregs were capable of suppressing Teff proliferation *in vitro*. Remarkably, human edTregs were able to substantially ameliorate xenogeneic GvHD caused by human CD4+ Teff cells in immunodeficient NOD mice. In addition, the investigators showed that adoptive transfer of antigen-specific edTregs from TCR transgenic 2D2 mice resulted in a reduction of CD45+CD4+ T cells in the EAE model of Multiple Sclerosis (MS), as compared to polyclonal edTregs. Finally, the authors were able to recapitulate their successful technique with human-derived antigen-specific edTregs. These cells were able to inhibit, *in vitro*, the proliferation of Teff cells with the same TCR specificity as well as with different specificities. Importantly, edTregs could be expanded 48-fold in 14 days, underlining their translational feasibility for the clinic.

In an effort to generate a well-defined and uniformly functional antigen-specific Treg population, Tenspolde et al., have turned their efforts into generating chimeric antigen receptor (CAR) Tregs **(** **Figure 1** **)**. While there are a few FDA approved CAR T-cell therapies in cancer treatment, CAR Tregs have not been used in the clinic yet. With their off-the-shelf usability and customized design, generating CAR-Tregs to treat T1D is a very promising avenue, especially in terms of avoiding off-target systemic suppression. Tenspolde et al., have carefully selected insulin-specific (a well-known autoantigen in T1D (31)) scFvs that showed the strongest binding to insulin, using a phage display library (32). Next they transduced CD4+ Teff cells with a CD28/CD3 second generation CAR construct that contained the insulin-specific scFvs, and a Foxp3 sequence, leading to the reprogramming of CD4+ T cells into insulin-specific Tregs (CAR-cTregs). A T cell hybridoma cell line that was transduced with the CAR constructs successfully expressed a GFP reporter downstream of an NFAT-sensitive IL-2 promoter after insulin stimulation, demonstrating the functionality of the design. Moreover, CAR-cTregs were phenotypically and functionally similar to natural Tregs: they proliferated in the presence of insulin and inhibited the proliferation of allospecific CD8+ Teff cells (32). However, although these CAR-cTregs were unable to prevent progression to diabetes in NOD females, they could still be detected up to 17 weeks following the adoptive transfer, constituting 2-4% of all splenic Tregs (32). This study is the first proof of concept for using CAR technology to convert Teff cells to Tregs, in an attempt to treat T1D.

As promising as all of the therapeutic advancements in Treg adoptive transfer might be, a caveat and a long term concern is the potential of these cells to convert back into autoreactive Teff cells. This is a major issue given that these cells are present in high numbers, and thus thorough long term monitoring to make sure that these cells remain true to their new identity will be critical before moving to clinical trial protocols. Altogether, Treg-based interventions designed to restore self-tolerance in T1D and other autoimmune diseases carry great hope due to their proven capacity to block disease progression in many animals models, their highly specialized and multifaceted immune suppressive functions and the emerging capacity to design these live drugs in ways that assure target specificity, optimally tailored functionality, and durability. Moreover, the potential risk associated with *ex vivo* engineered Tregs is mitigated by the capacity to extensively characterize and test the properties of these cells before they are re-introduced in the patients.

## Novel Methods to Promote Immune Regulation *In Situ*

There are a lot of reasons why many of the past and current therapeutic approaches that seem promising at first pre-clinically ultimately fail to prove efficacious in clinical trials. One such classic approach, that uses auto-antigen presentation to induce immune tolerance has been thoroughly examined for a variety of autoimmune diseases, including T1D (33, 34). Insulin is one of the main pancreatic auto-antigens targeted by T cells in T1D, and tolerance induction towards insulin showed promise in young NOD mice and in the transgenic mouse model, RIP-LCMV (35–37). However, translating these results into the clinic has been challenging (38). Hence converting this and other existing approaches using more refined drug presentation can plausibly overcome bench-to-bedside barriers. The integration of nanotechnology and biomaterials in immunotherapy holds great promise for such improved efficacy **(** **Figure 1** **)**. Precise cell targeting, delivery and controlled release of drugs, and suppression and/or activation of select aspects of the immune system are just a few of the potential strengths of such immunomodulatory agents (39, 40). In 2019, Dul *et al*., have shown that gold nanoparticles (AuNPs), covalently attached to PI_C19-A3_, a proinsulin peptide **(** **Figure 1** **)**, were extensively internalized by Langerhans cells after injection into *ex vivo* human skin, demonstrating that antigen-presenting cells can successfully uptake these nano-complexes (41). Gold itself is known to possess anti-inflammatory capabilities, whereas PI_C19-A3_ was previously shown to have an immunosuppressive effect on autoreactive CD4+ T cells (42). Importantly, monocyte-derived dendritic cells (moDCs) treated with AuNPs showed reduced ability to stimulate proliferation of naïve, but not memory, T cells, suggesting that this treatment may be more useful for the priming stage of the disease where immature DCs can promote Tregs. These promising results have led to test an AuNP-peptide formulation in an ongoing clinical trial (NCT02837094).

An alternative approach to induce a tolerogenic milieu by targeting DCs focused on activating the aryl hydrocarbon receptor (AhR), which was shown to impart tolerogenic properties to DCs, subsequently promoting the differentiation of naïve CD4+ T cells into Tregs (43, 44). Based on this premise, the authors generated gold-NPs covered with the AhR ligand, 2-(1′H-indole-3′-carbonyl)-thiazole-4-carboxylic acid methyl ester (ITE), and proinsulin (NP_ITE+Ins_) **(** **Figure 1** **)**. NP_ITE+Ins_ were able to suppress the development of spontaneous T1D in NOD mice. Moreover, T-bet and RORγT levels were reduced while Foxp3 expression was upregulated in pancreatic lymph nodes, indicating decreased presence of proinflammatory effector cells (Th1 and Th17) and an increase in Tregs. Splenic DCs from NOD mice that were stimulated with LPS showed a reduction in major histocompatibility complex (MHC II), CD40, CD86 and IL-12a and IL-6 levels, while upregulating anti-inflammatory IL-10. Similar tolerogenic characteristics were observed in human moDCs that became less potent in stimulating IFN-γ production by GAD-specific CD4+ T cells after treatment with NP_ITE+GAD_. Moreover, DCs that were incubated with NP_ITE+BDC2.5p_ showed reduced capability at inducing proliferation and cytokine production of TCR transgenic islet antigen-specific BDC2.5 T cells, while FoxP3+ CD4+ T cells were expanded. The authors demonstrated that the underlying mechanism for creating tolerogenic DCs was achieved by inhibition of NF-kB signaling in DCs, through AhR-mediated upregulation of SOCS2, successfully reestablishing antigen-specific tolerance (45).

An important breakthrough in the field of *in situ* induction of immune regulation came from Dr. Santamaria’s laboratory where they generated NPs coated with disease-related peptides bound to MHCII (46). Treatment of NOD mice, and humanized mice engrafted with patients’ lymphocytes, with these NP complexes promoted the differentiation of autoreactive T cells into antigen-specific regulatory CD4+ T cell type 1 (Tr1)-like cells **(** **Figure 1** **)**, and contributed to the development of disease-suppressing regulatory B cells (Breg). Disease reversal was achieved without negatively affecting systemic immunity. In another novel approach, Ag-associated carboxylated biodegradable poly(lactide-co-glycolide) nanoparticles (Ag-PLG) **(** **Figure 1** **)** restored tolerance in NOD.SCID mice that were adoptively transferred with diabetogenic Ag-specific CD4+ and CD8+ T cells (47). The authors showed that treatment with Ag-PLG nanoparticles affected autoreactive T cell trafficking. Treated mice had an intact pancreas architecture with the few T cell infiltrated areas mostly composed of Foxp3+ Tregs, when compared to mice treated with a non-diabetogenic antigen. PD-1 and CTLA-4 were involved in imparting protection against T1D in Ag-PLG treated mice. Furthermore, the authors demonstrated that Ag-PLG treatment expanded the peptide-specific Treg population among the adoptively transferred T cells (47). Most importantly, the induced tolerance was shown to be Ag-specific and PLG particles can carry several diabetogenic peptides simultaneously, which can be useful when numerous self epitopes are eliciting an autoimmune response, or if the exact auto-antigen is not known.

An interesting novel delivery vehicle for immunotherapeutic payloads are genetically modified *Lactococcus lactis* (*L. lactis*) bacteria, which are safe for consumption **(** **Figure 1** **)**. These live medicines are administered orally and exert their activities in the gut, an organ that likely plays a critical role in T1D etiology through mechanisms involving diet, the gut microbiota an gut barrier integrity. Changes in gut biology enables local activation of islet-reactive T cells and/or mucosal-associated invariant T cells (48–50). To induce auto-antigen based tolerance and Tregs, Dr. Mathieu’s group generated an *L. lactis* that secreted GAD65 (51) or proinsulin (52) together with IL-10 and a low dose of anti-CD3 mAb. This combination successfully reversed disease in NOD mice, which was associated with expansion of antigen-specific Foxp3+ Tregs. Based on these results, a clinical trial is currently underway (NCT03751007). This approach also highlights the importance of directing therapies towards the organs involved in pathophysiology, and, as such, there is great interest in developing technologies to localize treatments in the pancreas.

While an *in situ* approach to expand antigen-specific Tregs has the advantage of being less labor-intensive and likely more cost-effective than *ex vivo* engineering, a concern may be that the targets are less directly controllable due to the diverse and variable states of endogenous immune cell populations and ongoing responses in human subjects.

## Conclusions

With no currently available therapeutic interventions to treat the underlying pathophysiology of T1D, this disease continues to pose a weighty impediment both medically and financially. Even with rigorous monitoring and regulation of blood glucose, many patients still suffer from a wide range of debilitating clinical manifestations such as atherosclerosis and thrombotic events, nephropathy, neurocognitive decline, neuropathy and retinopathy (4), emphasizing the urgent need for innovative immunotherapies. Hence, emerging clinical interventions could potentially become tomorrow’s cure for T1D. Tregs which are known to be an Achilles heel in many T1D patients due to their low numbers and/or impaired functionality, constitute an attractive therapeutic target. More specifically, expanding this immunoregulating, tolerance-inducing population of cells in patients is a sough-after clinical goal. One approach would be to adoptively transfer *ex vivo* expanded Tregs back into the patient. In fact clinical trials in kidney transplantation showing improved clinical outcomes in some patients suggest that transfer of autologous Tregs can be promising across multiple immune-dysfunctional conditions (53–55). In addition, modifying the Tregs *ex vivo* before transplanting them back into the patient may increase efficacy and avoid potential systemic immunosuppression. The first in-human autologous CAR-Treg cell therapy, developed by Sangamo Therapeutics, was recently authorized for end-stage renal disease (ESRD) patients. The idea is that the CAR-Tregs will recognize the donor’s HLA-A2 molecule and localize within the transplant and induce immunosuppression, thus preventing kidney rejection. There is an extensive list of pre-clinical studies to support it that showed promising results both *in vitro* and *in vivo* (56–59).

The era of bioengineering, encompassing nanotechnology, biomaterials and more, is not only adding another layer of potential advancement and precision to some of the most promising therapies that currently lack significant efficacy in humans, but is also more practical in use than *ex vivo* cell engineering. As described in this review, novel delivery methods and materials may allow for precise targeting of cell types, such as Tregs that need to be stimulated and enhanced in order to restore a tolerizing milieu in target tissues. For example, targeted antigen-specific therapies such as NPs covered with the AhR ligand, ITE, and proinsulin that were developed by AnTolRx, became a licensed therapy that was recently acquired by Pfizer, emphasizing their potential. Given their promising results, AnTolRx is working on adapting their NPs to other autoimmune disease such as MS, rheumatoid arthritis, psoriasis and more. Altogether, both frontiers, *ex vivo* and *in situ* Treg expansion and enhancement, despite each having their own advantages and limitations, carry great promise as emerging, perhaps soon to be implemented in the clinic, therapeutics.

## Author Contributions

HD and VV-S developed the concept for this review. VV-S, AJ, and JH-E researched the literature and wrote the manuscript. VV-S and HD designed the figure. HD edited the manuscript. All authors contributed to the article and approved the submitted version.

## Funding

The authors are grateful for funding from the National Institute of Diabetes and Digestive and Kidney Diseases (NIDDK, www.niddk.nih.gov) 1R01 DK-102911 (HD).

## Conflict of Interest

The authors declare that the research was conducted in the absence of any commercial or financial relationships that could be construed as a potential conflict of interest.
